# Multiscale correlative tomography: an investigation of creep cavitation in 316 stainless steel

**DOI:** 10.1038/s41598-017-06976-5

**Published:** 2017-08-04

**Authors:** T. J. A. Slater, R. S. Bradley, G. Bertali, R. Geurts, S. M. Northover, M. G. Burke, S. J. Haigh, T. L. Burnett, P. J. Withers

**Affiliations:** 1 Henry Moseley X-ray Imaging Facility, School of Materials, The University of Manchester, Manchester, M13 9PL UK; 2 School of Materials, The University of Manchester, Manchester, M13 9PL UK; 3 Thermo Fischer Scientific, FEI Company, Achtseweg Noord 5, Bldg, 5651 GG Eindhoven, The Netherlands; 4 Department of Engineering and Innovation, Materials Engineering, The Open University, Milton Keynes, MK7 6AA UK

## Abstract

Creep cavitation in an ex-service nuclear steam header Type 316 stainless steel sample is investigated through a multiscale tomography workflow spanning eight orders of magnitude, combining X-ray computed tomography (CT), plasma focused ion beam (FIB) scanning electron microscope (SEM) imaging and scanning transmission electron microscope (STEM) tomography. Guided by microscale X-ray CT, nanoscale X-ray CT is used to investigate the size and morphology of cavities at a triple point of grain boundaries. In order to understand the factors affecting the extent of cavitation, the orientation and crystallographic misorientation of each boundary is characterised using electron backscatter diffraction (EBSD). Additionally, in order to better understand boundary phase growth, the chemistry of a single boundary and its associated secondary phase precipitates is probed through STEM energy dispersive X-ray (EDX) tomography. The difference in cavitation of the three grain boundaries investigated suggests that the orientation of grain boundaries with respect to the direction of principal stress is important in the promotion of cavity formation.

## Introduction

Hierarchical structures underpin key processes in materials science, chemical engineering, biology and geology. In all of these hierarchical materials the performance depends critically on features at length scales from the macroscale to the nanoscale. For instance, in the geological sciences, the multiscale porosity exhibited in hydrocarbon containing carbonate rocks determines the efficiency of extraction of oil and gas reserves^1–3^. Additionally, features at one length scale often directly affect material features at another length scale. For example, nanoscale precipitates in steels can affect the microscale formation of cracks and hence influence the macroscopic deformation and resulting lifetime of an engineering component when exposed to repeated stress or harsh environmental conditions^4, 5^. However, no one technique can probe spatial and chemical information at sub-nanometre resolution for macroscopic volumes in a reasonable timescale. As a consequence, there is great interest in combining multiple imaging techniques with different resolutions, to link macro-, micro- and nanoscale features^6–8^.

Conventionally, three dimensional (3D) microstructural investigations covering a wide range of length scales have involved multiple instruments looking at different regions taken from the sample material^6–8^. Although valuable, these uncorrelated data sets can fail to identify key relationships between nanoscale structures and macroscopic features. Deeper insights can be gained using a correlative approach^9^, in which the same region of interest (RoI) is probed at different length scales, as is presented in this paper and termed multiscale correlative tomography, or with different techniques at the same length scale^10, 11^, which we term multifaceted correlative tomography. 3D data acquired using a number of techniques are then spatially registered in order to provide a wealth of structural and chemical information at the same sample location. To date, multiscale correlative tomography has been applied to the spatial correlation of data from X-ray computed tomography (CT) and serial section electron tomography using dual beam focused ion beam (FIB) scanning electron microscopes (SEMs)^9, 12^, limiting the resolution to tens of nanometres, although serial section FIB-SEM can reach lateral resolutions of a few nanometres in optimal circumstances^13^.

**Figure 1:**
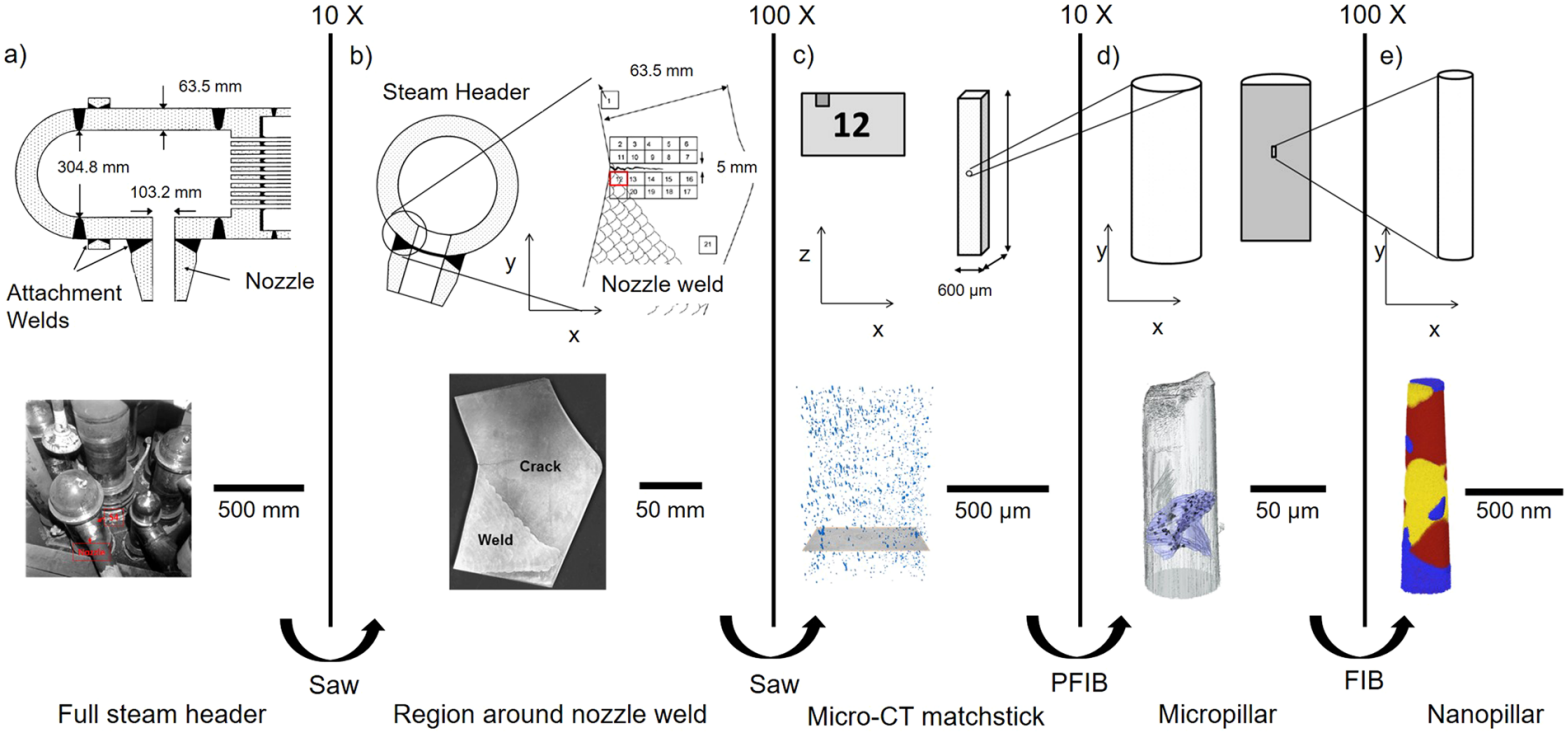
Illustration of the methodology applied in the study of a weld region of an AGR power station steam header, showing schematics and images of (**a**) the full steam header, (**b**) a section cut from the header, (**c**) a matchstick imaged with micro-CT, (**d**) a micropillar containing cavitated grain boundaries imaged with nano-CT and (**e**) a nanopillar taken from a grain boundary imaged with STEM-EDX tomography.

In order to extend the resolution limits of 3D imaging it is necessary to include information obtained from higher resolution techniques such as transmission electron microscope (TEM) based electron tomography^14, 15^ or atom probe tomography (APT)^16^. These techniques not only offer high lateral resolution morphological information but can also provide chemical information at nanometre resolution^17–19^. Furthermore, by adopting a correlative approach, one can mitigate an inherent disadvantage of such high resolution techniques in that they focus on a very small region which may or may not be representative; by providing larger scale contextual information one can ensure that the material analysed at the nanoscale is characteristic of the features of interest. By incorporating transmission electron or atom probe tomography it is possible to extend the tomography workflow to nanometre resolution, allowing spatial correlation of volumes from metre length scales, down to less than 100 nm (Fig. 1).

In this paper, a multiscale tomography workflow is applied to investigate creep cavitation in a sample of Type 316 stainless steel taken from an advanced gas-cooled reactor (AGR) steam header (Fig. 1a). Creep cavitation and subsequent reheat cracking are key failure mechanisms in the lifetime of welded steel sections, with a total of 261 reheat cracking incidents found in AGRs up to 2004^20^. The critical region is the heat affected zone in which creep cavitation failure occurs, driven only by the weld residual stresses, a prone microstructure and prolonged exposure to temperatures of around 550 °C^21^.

Significant information about the extent and causes of creep cavitation around the steam header crack investigated in this study has previously been obtained over larger volumes on a statistical basis using small angle neutron scattering (SANS)^20^ and microscale X-ray CT^21^. The growth of cavities is largely driven by hydrostatic stress, with the magnitude of the residual stresses at a particular grain boundary playing a large role in the number and size of cavities, as previously determined by SANS of the macroscale crack^20, 22^. Grain boundaries further from the principal cracks display fewer and smaller cavities. The region investigated in this study is from a heavily cavitated region in close proximity to the crack mouth. The angle of the grain boundary to the direction of the residual stress is also thought to play a role in cavitation, microscale X-ray CT of the larger region of interest (region 12 in Fig. 1b and c) found cavitated grain boundaries were primarily oriented at approximately 45° to the principal crack^21^. It has been suggested that cavities on transverse boundaries should grow more quickly due to their orientation to the direction of principal stress^23^, with dislocation pile-up suggested as a mechanism for the accumulation of the required stress for cavity nucleation^24^. Additionally, it has been hypothesized that grain boundary sliding contributes to cavity nucleation and growth^23, 25^, hence inclined boundaries (such as those observed in the previous microscale CT) should nucleate cavities more readily than transverse boundaries. In this model grain boundary precipitates act to restrict grain boundary sliding and local triaxial stresses of a large magnitude develop at grain boundaries, resulting in cavitation. However, cavitation has been observed on transverse boundaries with no inclination, suggesting that in some systems cavitation occurs without contributions from grain boundary sliding^24, 26^.

Previous studies have also found that intergranular carbides play a key role in the nucleation of cavities, acting to pin vacancies and cause their coalescence into larger cavities^21, 22^. It has been shown that the formation of intergranular carbides is highly dependent on grain boundary misorientation; random high-angle misorientations result in a greater degree of carbide precipitation than twin boundaries, with coherent twin boundaries found to be the only type of boundary displaying no precipitation^27, 28^. Additionally, an increase in the deviation away from zero misorientation or high coincidence misorientations is also associated with a change in carbide shape from plate-like to triangular, which has been shown to correlate to a greater degree of cavitation^27^. The growth of cavities has also been linked to whether they sit at an austenite-austenite or austenite-ferrite-austenite boundary, with the latter resulting in preferential cavity growth and a lenticular cavity morphology^29^. A multi-scale study of a different area to that characterised in this study, but taken from the same larger region (region 12 in Fig. 1b and c), previously identified carbide and ferrite precipitates associated with a single cavitated grain boundary^21^.

In this study, a multiscale tomography workflow is employed to investigate how grain boundary properties affect localised cavitation. 50 nm resolution X-ray CT is employed to characterise the size and shape of cavities on three grain boundaries, FIB-SEM serial sectioning and electron backscatter diffraction (EBSD) mapping is used to determine the extent of precipitation and the crystallographic misorientation at each grain boundary and, finally, STEM-EDX tomography is used to map the chemical distribution within precipitates at a precisely located site on one of the cavitated grain boundaries. Each of the applied imaging techniques is performed within the overall volume of the X-ray CT, with the location of each subsequent step chosen at a specific location from the previous larger scale volume.

## Results

### Characterisation of Cavitation via Nanoscale X-ray Tomography

**Figure 2:**
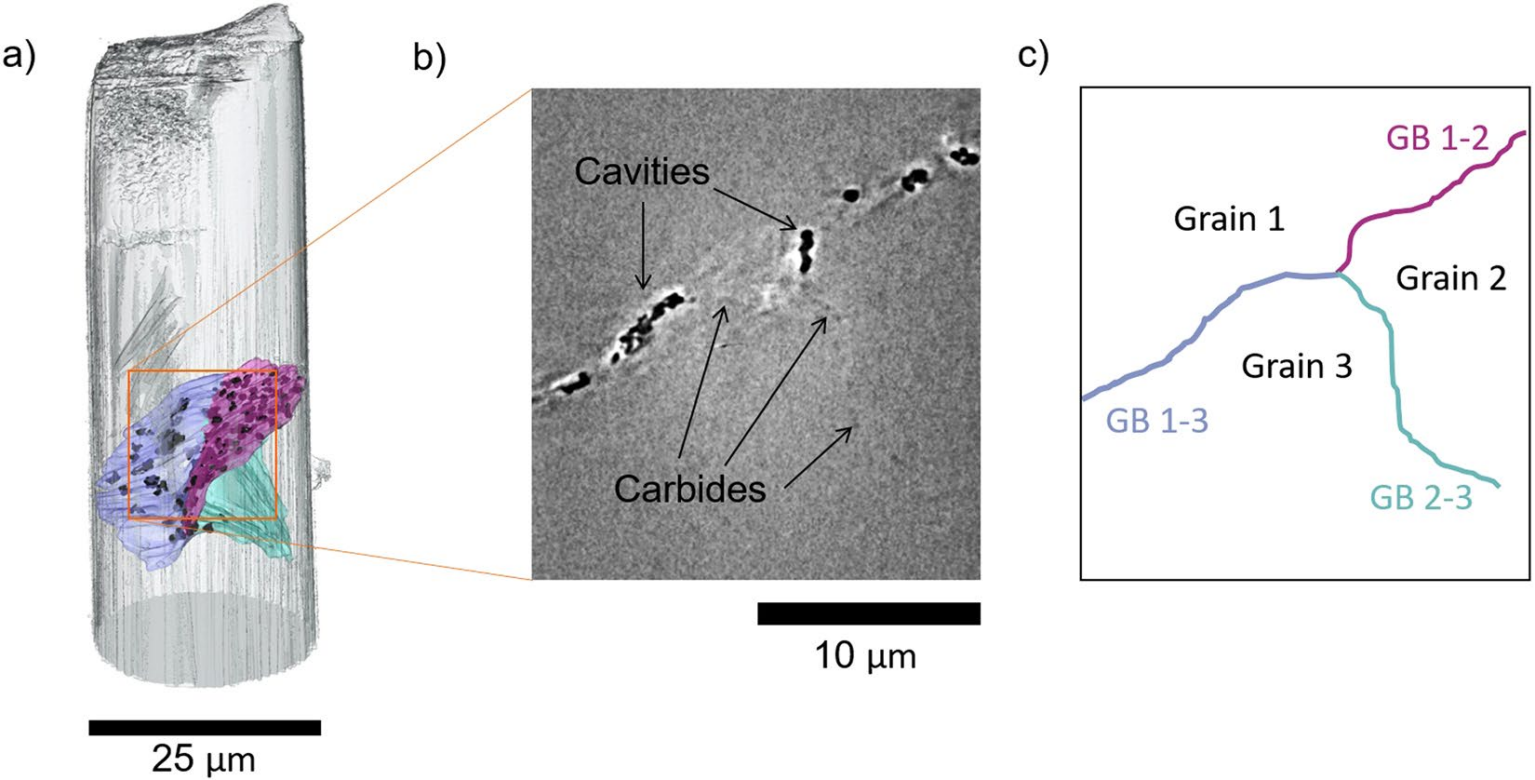
X-ray nanotomography of the micropillar of the ex-service Type 316 stainless steel containing a cavitated grain boundary. (**a**) Volume visualisation displaying an isosurface rendering of the outer surface of the pillar, with traces along the three grain boundaries (coloured blue (grain boundary 1–3), magenta (grain boundary 1–2), and turquoise (grain boundary 2–3)) and the surface of the segmented cavities (black). (**b**) Slice through the X-ray nanotomography reconstruction displaying the three grain boundaries and the intensity changes associated with cavities and intergranular carbides. (**c**) Schematic of the grain boundaries in a 2D slice based on the intensities observed in (**b**).

The presence of three grain boundaries within the micropillar was revealed by X-ray nanotomography, two decorated with cavities and one visible only through small intensity differences arising from large intergranular carbides (Fig. 2). The three grain boundaries meet at a triple junction in the middle of the pillar, which can be visualised in three dimensions through manual tracking of intensity variations due to the intergranular carbides (Fig. 2). The nano-CT data highlights the location and morphology of individual cavities along the cavitated grain boundary. The carbides that are visible as slight changes in intensity along the grain boundary are also present along the cavitated grain boundary, but their morphology cannot be determined due to the low X-ray contrast.

**Figure 3:**
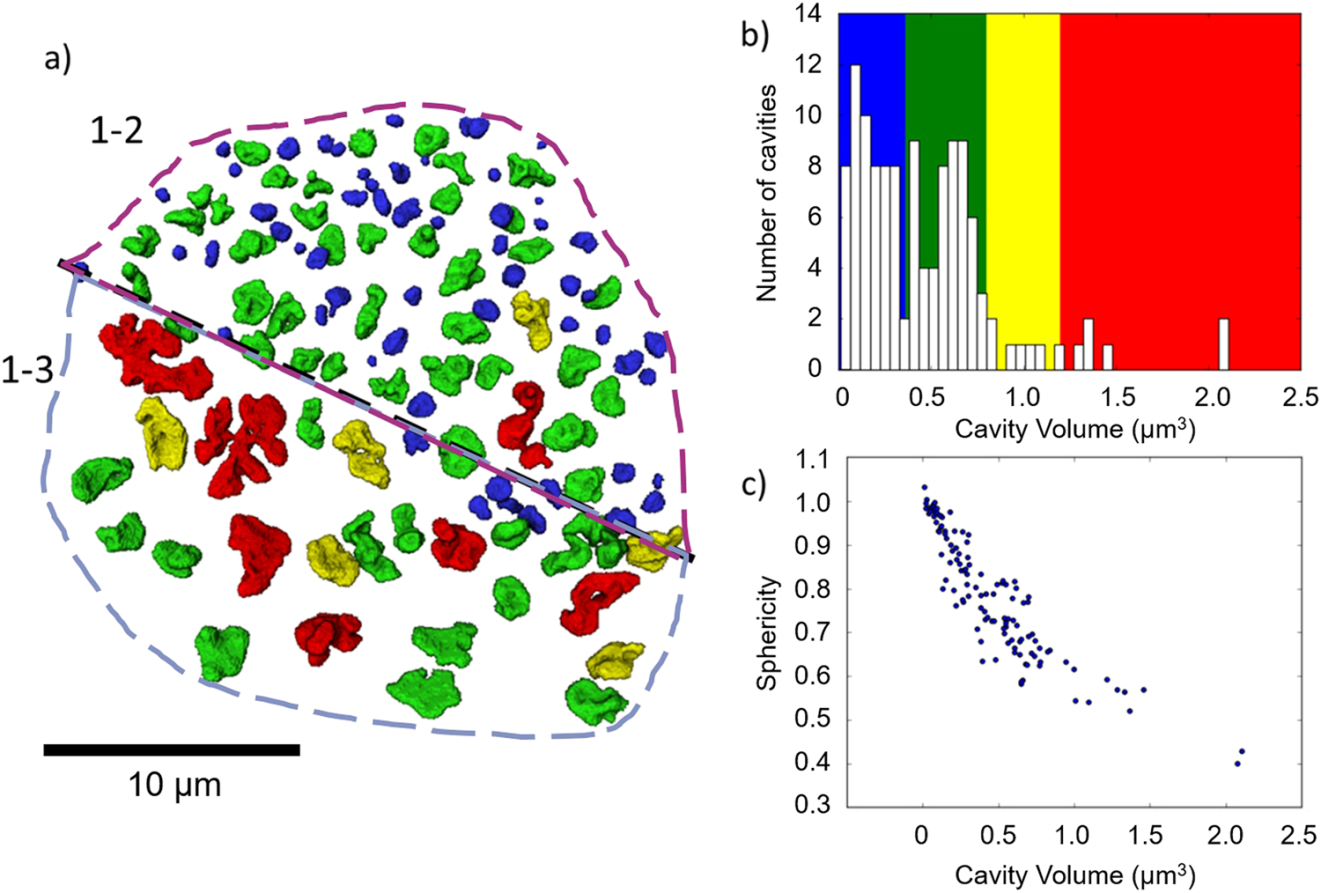
(**a**) Volume rendering from X-ray nanotomography data showing the presence of cavities at grain boundaries 1–2 and 1–3 with colour mapping based on the volume of cavities. (**b**) Histogram of cavity volumes displaying the colour map associated with the volume rendering in (**a**). (**c**) Scatter plot of sphericity of cavities vs volume.

It is evident that there is a significant difference in the size and morphology of cavities between the two cavitated grain boundaries (Fig. 3a). On grain boundary 1–2 (magenta in Fig. 2) the cavities are primarily smaller and more spherical, whereas for grain boundary 1–3 (blue in Fig. 2) the cavities are larger in volume and have a more complex morphology. The size and morphology of the cavities seem to be related, with the larger cavities showing a greater departure from a spherical morphology (Fig. 3c). The difference in cavity size and morphology between the two boundaries suggests that there is an underlying difference in the grain boundary properties affecting cavity growth or coalescence. Larger cavities show a lenticular shape in general, with largest dimensions along the grain boundaries, suggesting that the cavities extend in size primarily along the grain boundaries. Irregular ‘lobes’ can also be seen on larger cavities that may be indicative of cavity coalescence.

Previous studies have indicated that cavitation at grain boundaries is primarily governed by the orientation of the boundary to the residual stress field^21, 30^. For this reason, the angle between each grain boundary plane and the direction of principal stress was measured here. The cavitated grain boundaries (grain boundaries 1–2 and 1–3) were found to be at angles of approximately 60° to the direction of principal stress. In comparison, the angle between the non-cavitated grain boundary (grain boundary 2–3) and the direction of principal stress was measured as approximately 30°.

### Characterisation of Grain Boundaries by FIB-SEM and EBSD Analysis

**Figure 4:**
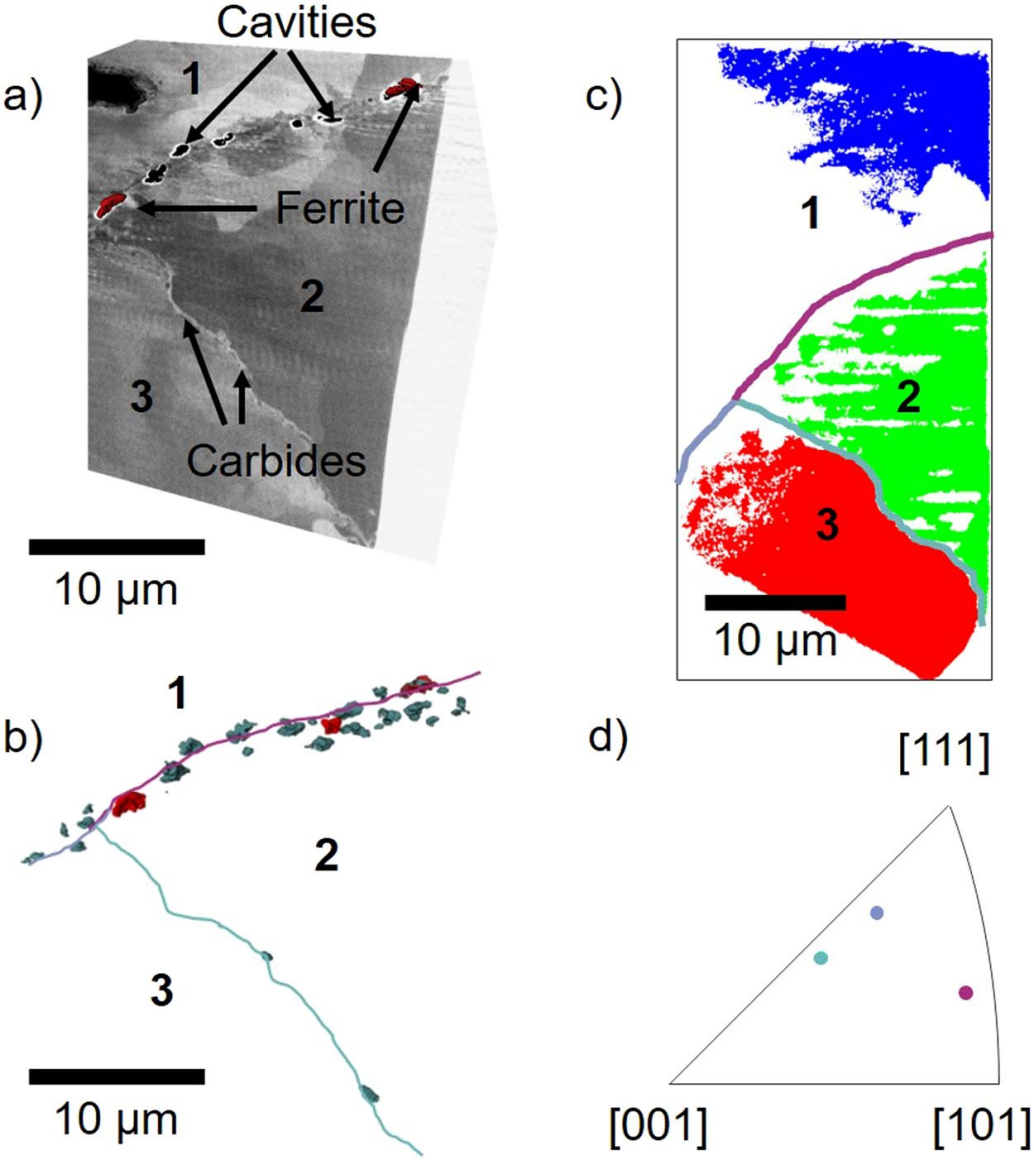
(**a**) Slice through the FIB-SEM volume showing the presence of carbides, ferrite and cavities for the three grain boundaries. The cavities associated with ferrite precipitates are shown in red. (**b**) Volume rendering of segmented cavities (turquoise) in the FIB-SEM volume, displaying those cavities associated with ferrite (red). The three grain boundaries are annotated in 2D (GB 1–2 is magenta, GB 1–3 is blue and GB 2–3 is turquoise). (**c**) The three grains, segmented from the EBSD data, labelled as blue (grain 1), green (grain 2) and red (grain 3). The three grain boundaries are annotated. (**d**) Inverse pole figure showing the axis of misorientation of the three grain boundaries.

FIB-SEM serial sectioning was employed to reveal the extent of precipitation at each of the analysed grain boundaries and to identify cavities associated with ferrite precipitates. From the secondary electron images it is clear that all three grain boundaries contained within the micropillar are decorated with secondary phase particles (Fig. 4a). The larger bright regions have previously been identified as ferrite using electron diffraction in an earlier study^21^ and the small darker particles have been identified as carbides by their chemistry. Four cavities within the serial sectioning volume are found to be associated with identifiable ferrite regions (Fig. 4b), but all cavities display a complex morphology with some aspect of lenticular growth that is not associated with the presence of ferrite. Cavities are clearly elongated along the grain boundaries, although they also show growth in to grains on either side of the boundary.

EBSD mapping was performed in order to determine the crystallographic misorientation across each grain boundary. EBSD analysis confirms the presence of three distinct boundaries within the sample volume studied (Fig. 4c), as inferred from the X-ray CT data. The EBSD analysis also confirmed the presence of bcc-iron in small regions at the grain boundaries. However, EBSD is unable to index large regions of the sample, possibly due to damage in the material caused by the non-optimum conditions﻿ i﻿n﻿ the ion milling of a ﻿c﻿ylinder.

The misorientation of the three grain boundaries was extracted from the EBSD data in order to explore whether misorientation affects the tendency for cavitation. The misorientations are summarised in Table 1, with the axis of misorientation displayed in Fig. 4d. None of the grain boundaries have a misorientation close to that of a low order coincident site lattice (CSL) boundary.

**Table 1:** Properties of each of the three grain boundaries.

Grain boundary	Cavitated?	Angle to direction of principal stress	Crystallographic Misorienation
1–2	Yes	62°	58°
1–3	Yes	63°	45°
2–3	No	31°	35°

### Mapping the Chemistry of Phase Boundaries via STEM-EDX tomography

In order to better understand the growth of each grain boundary phase, a nanoscale volume of interest (VoI) was identified to map changes in chemistry at the boundary between each phase. A “slab-like” morphology was chosen so as to contain all three grain boundaries, relaxing the requirement for precise vertical positioning of the slab. This was lifted out from the micropillar and attached to a “pronged” half-moon TEM grid before a 2 μm block was removed. It was then possible to determine the optimal location for subsequent extraction of a suitable STEM tomography nanopillar of length greater than 1 μm and diameter 200–300 nm using annular milling in the FIB-SEM. The final location of the nanopillar within the micropillar can be accurately determined through correlation of the X-ray CT data and SEM images acquired at a number of steps during the FIB milling process (see the Supplementary Information for details of this correlation workflow). The use of SEM images as a correlative step are required as features in the STEM-EDX volume cannot be directly correlated to features in the X-ray CT volume.

**Figure 5:**
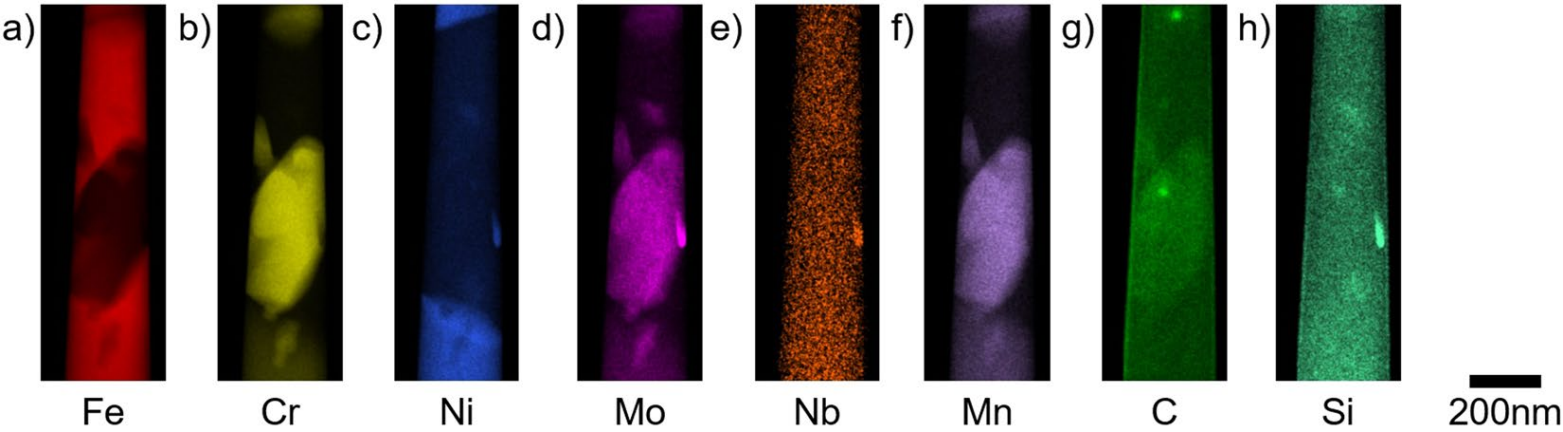
STEM-EDX elemental maps of the STEM needle displaying the intensity of X-ray counts associated with the (**a**) Fe Kα, (**b**) Cr Kα, (**c**) Ni Kα, (**d**) Mo Lα, (**e**) Nb Kα, (**f**) Mn Kα, (**g**) C Kα and (**h**) Si Kα peaks. The austenite phase at top and bottom is associated with Fe, Ni and to a lesser extent Mo and Mn, the ferrite phase is predominantly Fe with a small amount of Ni and Cr, the carbides contain Cr, Fe, Mo, C, Ni and Mn, the G-phase precipitate contains Nb, Ni, Mo and Si. The surface of the needle has high carbon and silicon intensities that are associated with organic contamination, primarily due to long exposure under the electron beam.

In order to locate the various nanoscale secondary phases, STEM-EDX tomography was performed on the nanopillar. This technique also allowed examination of the interfaces between the different phases to provide information on the growth of each phase. Previous characterisation of this material^21^ has identified the presence of secondary phases of ferrite, carbides and G-phase decorating similar cavitated boundaries within a different region of the same matchstick sample. STEM-EDX elemental analyses of the nanopillar prepared here provides evidence of the same secondary phases, sandwiched between two austenitic regions (Fig. 5). The prepared nanopillar also fully encapsulates a small (~50 nm diameter) carbide situated at an austenite-ferrite interface, allowing a full morphological description of an individual precipitate.

**Figure 6:**
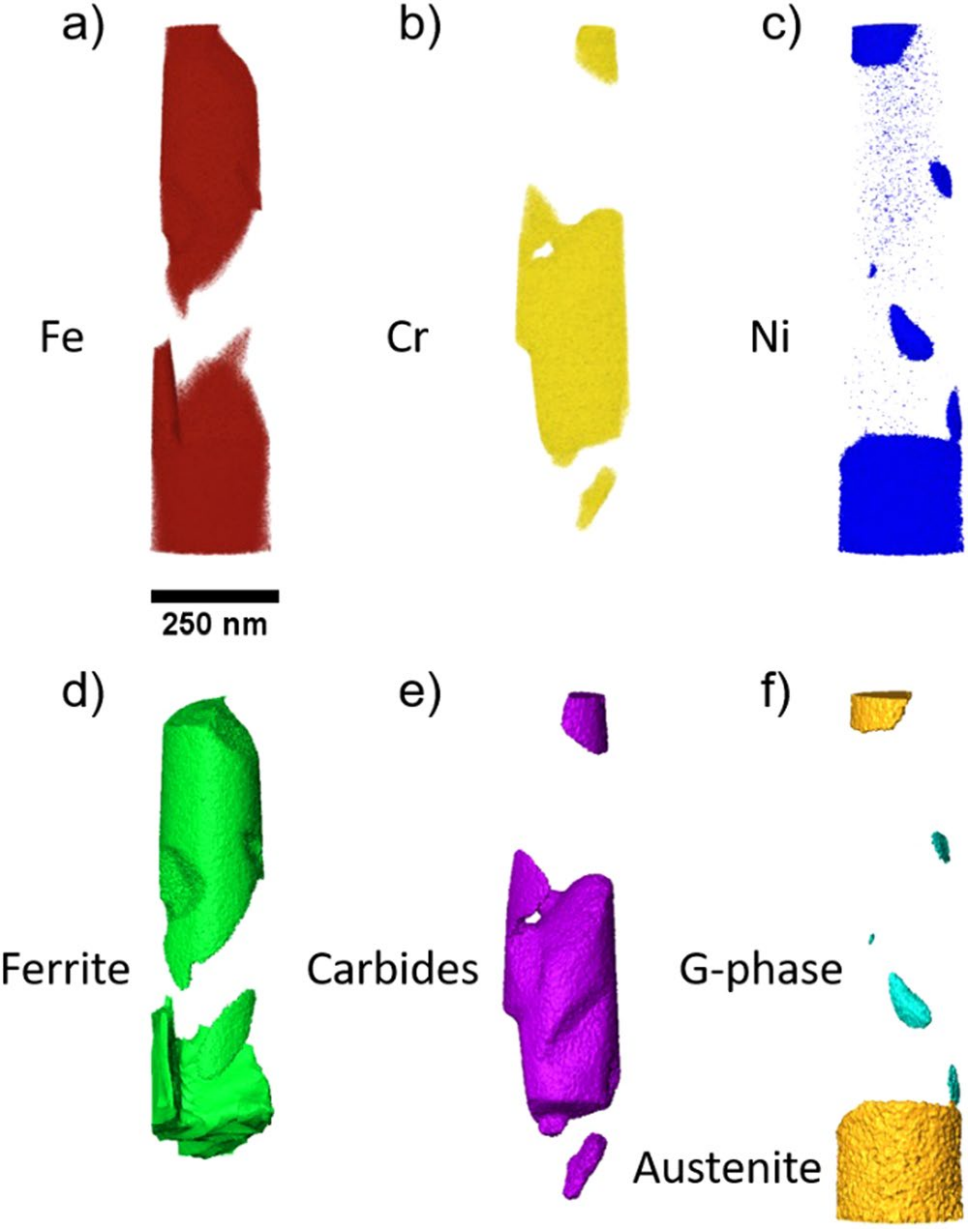
Visualisation of the three dimensional reconstruction of elemental distributions and the associated segmented phases. Volume visualisations of (**a**) the Fe Kα signal (red), (**b**) the Cr Kα signal (yellow) and (**c**) the Ni Kα signal (blue). Surface visualisations of the segmented phases showing the (**d**) ferrite phases (green), (**e**) carbide precipitates (purple) and (**f**) austenite (orange) and G-phase precipitates (cyan).

The reconstruction of STEM-EDX tomography tilt series involves the reconstruction of the X-ray intensity associated with individual elements, such that there is a separate reconstruction of the intensity distribution of the X-ray signal from each element^31^. Here, we have reconstructed the distribution of Fe, Cr and Ni in three dimensions (Fig. 6a–c), as these elements allow segmentation of the identified phases. The regions in which there is a high Fe intensity with a high associated Ni signal is associated with the austenite phase and that of a high Fe intensity with a much lower associated Ni intensity is associated with ferrite. Similarly, a high intensity in the Cr reconstruction corresponds to the carbide precipitates and a high intensity in the Ni reconstruction with no associated Fe intensity is associated to the G-phase. Through segmentation of the elemental data using these constraints the individual phases are segmented in three dimensions (Fig. 6d–f).

**Figure 7:**
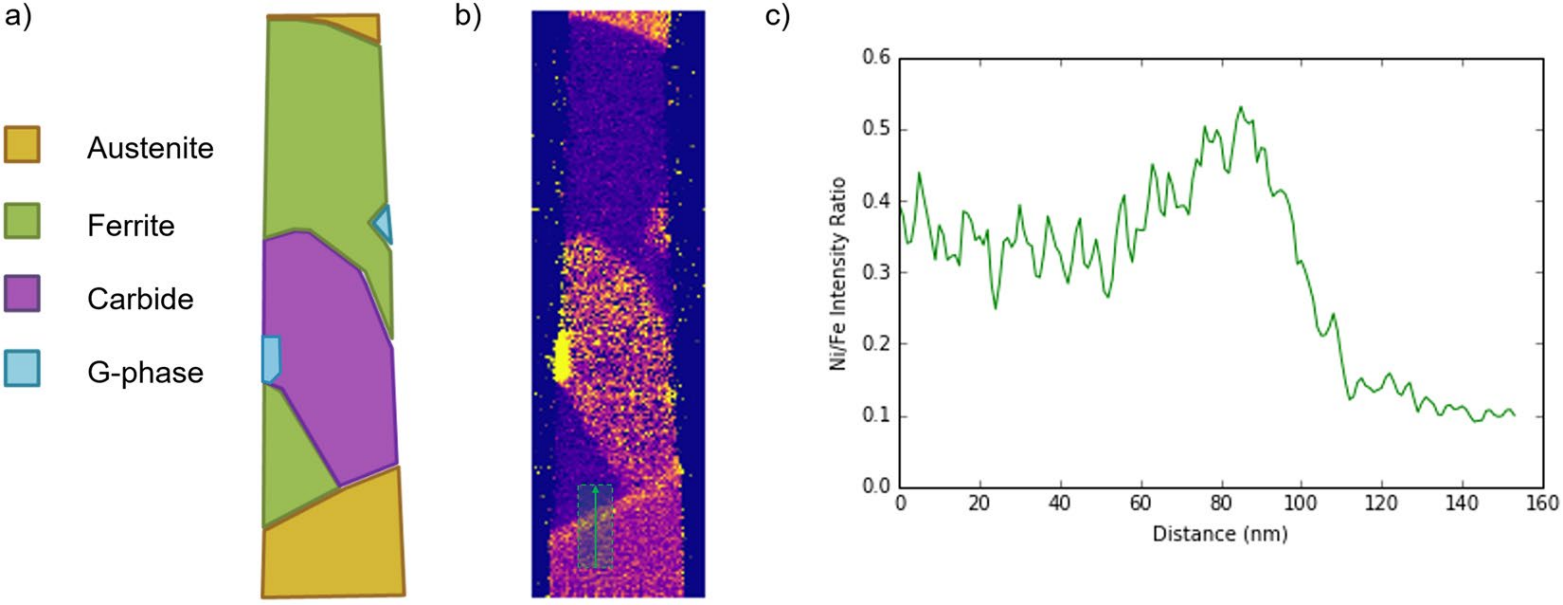
(**a**) Diagram displaying the different phases associated with the corresponding slice (**b**) through the STEM-EDX volume. (**b**) Slice displaying the ratio of Ni to Fe intensity through the centre of the STEM-EDX volume. An increased Ni/Fe ratio is clearly seen at the lower boundary between austenite and ferrite. (**c**) Line profile of the ratio of Ni to Fe intensity in the slice shown in (**b**), taken from the red region indicated in (**b**).

The three dimensional STEM-EDX reconstruction allows not only phase identification, but also a limited ability to map changes in composition within the sample volume. Here, we are interested in probing possible elemental segregation at the interfaces between different phases. From the STEM-EDX tomography volumes, the Ni intensity was divided by the Fe intensity to produce a volume displaying the ratio of Ni/Fe intensities (Fig. 7). The ratio volume shows increased Ni content (or depleted Fe content) at the lower austenite-ferrite boundary compared to within the austenite phase, as tentatively suggested previously^21^.

## Discussion

We have performed a multiscale tomography characterisation of three distinct grain boundaries to investigate the role of grain boundary properties and grain boundary phases on cavitation. There are clear differences in the extent of cavitation of each boundary, with one boundary showing no cavities, one displaying numerous small cavities and the other showing larger, less spherical cavities.

The fact that cavitation is observed on grain boundaries oriented so that the grain boundary plane is at approximately 60° to the direction of principal stress indicates that cavity nucleation and growth occurs due to a complex combination of stresses. The previous observations of Wahab *et al*.^30^ that the relative frequency of creep void formation in gas reformer tubes is highest at boundaries decorated with carbides and inclined at 40° – 70° away from the direction of the principal stress, and that of Burnett *et al*.^21^ that heavily creep cavitated boundaries are decorated with carbides and other precipitates and lie at approximately 45° to the apparent direction of the principal tensile stress, both suggest that shear at boundaries containing precipitates is conducive to the development of creep voids and that grain boundary sliding may play a significant role in void formation, as expounded in theoretical models of creep cavitation^21, 22, 24^. The present observation that boundaries at angles of 63° and 62° to the principal stress axis were cavitated but that one at an angle of 31° to the stress axis is consistent with Wahab *et al*.’s observation^30^ that at the mid-wall-thickness of a tube (i.e. where there is the highest constraint) most creep voids formed at boundaries themselves lying at 50° – 70° to the stress axis. The closer the boundary plane normal lies to the principal tensile stress, the higher the driving force for vacancy nucleation but the closer the boundary to 45° from the principal stress, the higher the driving force for grain boundary sliding. If we consider that nucleation and growth of cavities is due to a combination of these two factors, cavities might be expected to be able to form and grow more easily at boundaries that lie at an angle about 60° to the stress axis than one that lies at 31° to it.

Our analysis of the crystallographic misorientations across the grain boundaries revealed that all of the grain boundaries are high angle boundaries with no specific low-order CSL relationships. It has been suggested that the misorientation between neighbouring grains is a factor in carbide precipitation and therefore cavity growth^27, 28^. Analysis of only three boundaries could not provide conclusive evidence of this but differences in both the crystallographic misorientations and the orientation of the grain boundary plane with respect to the crystallography of each grain may have played a role in the differences in size and morphology of the cavities at the two cavitated grain boundaries; specifically through their effects on the nature of the carbide/matrix interfaces and hence their effectiveness in providing sources of dislocations or vacancies to enable sliding and cavitation to occur.

Each of the grain boundaries analysed were decorated to similar levels with both carbides and ferrite, suggesting that their formation is not dependent on the orientation of the boundaries to the direction of principal stress. It is thought that the presence of intergranular carbides acts to promote vacancy coalescence but it is clear that the presence of intergranular carbides does not in itself lead to cavitation at a grain boundary. Certainly, it is clear that intergranular carbides are present at grain boundaries both with and without cavitation. Similarly, we have found ferrite formation occurred both with and without associated cavities, suggesting that ferrite formation is not dependent on cavity nucleation. However, it is probable that grain boundaries decorated with carbides and ferrite show differences in cavity nucleation to undecorated boundaries, but no undecorated boundaries were observed in the small sample studied here.

There appears to be a clear difference in the cavities decorating the two cavitated grain boundaries, with one boundary possessing a greater degree of large non-spherical cavities. The formation of cavities may occur through either distinct steps of nucleation, followed by growth, or through simultaneously occurring nucleation and growth. In addition, growth may occur either through the continuous growth of individual cavities or through the coalescence of small cavities. Larger cavities appear to show lenticular profiles along grain boundaries, suggesting cavities grew in parallel to grain boundaries, and this observation was not related to the presence of ferrite bordering the cavity. This is in contrast to the observations of Warren *et al*.^29^ that cavities at austenite-austenite boundaries maintain a spherical morphology. Additionally, some larger cavities also displayed deviation away from a lens shape with pronounced ‘lobes’, which would be expected if growth occurs through coalescence. Grain boundary sliding could also play an important role in this context, promoting the coalescence of cavities. The differences in cavity size and morphology between the two grain boundaries could be due to capturing the same growth process at different stages, i.e. the boundary decorated with larger cavities experienced nucleation earlier in the lifetime of the steam header. Alternatively, nucleation may have occurred simultaneously on both boundaries (or is continuously occurring) and one grain boundary allows a greater rate of vacancy or cavity diffusion. In order to unequivocally characterise the cavity nucleation and growth mechanisms, observations of cavity growth along the lifetime of the steam header would be required.

From the STEM-EDX elemental mapping of the ferrite-austenite interface it is apparent that austenite in this region has a greater concentration of Ni compared to austenite away from the interface. This supports similar results from previous two dimensional mapping^21^ and provides evidence that this ferrite formed over the lifetime of the sample, rejecting Ni to the surrounding austenite. Whether the ferrite or cavities nucleate first remains unclear, although it may be possible that the irregular faceted morphology of cavities is due to secondary phases growing in to these cavities.

## Conclusions

This paper provides the first example of spatial correlation between X-ray tomography and STEM tomography datasets, together with a robust methodology, which represents the first step towards a correlative workflow between the two techniques. The methodology allows specific locations in a volume of interest to be prepared for STEM tomography studies.

A multiscale approach has allowed us to make the following conclusions about creep cavitation at a specific location in a Type 316 grade stainless steel steam header:

- The orientation of a grain boundary with respect to the direction of principal stress is an important factor in cavitation. In the three grain boundaries examined, cavitation occurs at grain boundaries oriented at ~ 60° to the direction of principal stress but not at one oriented at ~ 30°. This indicates that grain boundary sliding may be an important mechanism in cavity formation.
- Cavity growth does not occur uniformly for cavities on a single grain boundary. Larger grain boundary cavities are less spherical than smaller cavities, displaying irregular morphologies with the largest dimensions in the plane of grain boundaries. The irregular morphologies of large cavities suggest that grain boundary sliding may also play a role in the development of cavitation, aiding the coalescence of cavities.
- Grain boundary precipitates do not explicitly lead to cavitation. The three grain boundaries investigated here are all similarly decorated with precipitates but do not all display cavitation.
- There is greater Ni content at austenite-ferrite boundaries than in the austenite matrix, suggesting that grain boundary associated ferrite grows over the component lifetime, ejecting Ni in to the surrounding austenite matrix.

## Methods

### Sample

Sections 8 mm thick were cut from an ex-service steam header so as to include the header and nozzle weld. In particular, attention was focused on two adjacent slices of material machined from the steam header-nozzle weld (1C1/1) containing a ~30 mm deep crack used for the creep damage investigations, see Fig. 1b. The header was made from AISI Type 316H austenitic stainless steel; the composition of the as-cast header is given as 0.058 wt% C, 0.42 wt% Si, 1.61 wt% Mn, 0.009 wt% P, 0.02 wt% S, 17.33 wt% Cr, 11.29 wt% Ni, 2.31 wt% Mo, 0.12 wt% Cu, 0.04 wt% V, 0.041 wt% N, <0.07 wt% Co and <0.01 wt% Ti and Al^20^. The header had been removed from service after 65,000 hours of operation under internal pressure loading of 16 MPa at about 525 °C, following the discovery of extensive cracking around the nozzle region by ultrasonic inspection.

A 10 mm^2^ rectangular coupon, 8 mm thick, was extracted from a location before the crack mouth and the outer surface of the header (labelled 12 in Fig. 1b and c). A SiC circular saw was used to cut matchsticks of length of 0.74 mm and cross-section of 0.36 mm^2^ from the coupon of interest. A cavitated grain boundary was found in a volume of interest in one of the matchsticks previously imaged using microscale X-ray CT at the TOMCAT beamline at the Swiss Light Source^21^.

### Plasma-FIB

A FEI Helios Xe plasma-FIB was used to extract a pillar of approximately 25 µm diameter, at the location of the cavitated boundary, for subsequent nanoscale X-ray CT. Initially, the location of the cavitated grain boundary was confirmed through milling of a cross-section and ion-beam imaging. Subsequently, annular milling was used to prepare a pillar, using a milling current of 1.3 µA and two subsequent polishing steps of 180 nA and 59 nA. The pillar was attached to a liftout probe, undercut with a 59 nA beam and was then subsequently lifted out. The pillar was then attached to the tip of a copper pin using Pt deposition, before the liftout probe was detached. The ion-beam remained at an accelerating voltage of 30 kV throughout. The micropillar was produced so that its long-axis is perpendicular to the direction of the macroscale crack plane, meaning the long-axis of the pillar is parallel to the principal stress direction. The plasma-FIB provides a rapid method of machining volumes at the scales of tens or hundreds of micrometres, providing milling rates up to fifty times greater than conventional Ga+ ion beam milling^32, 33^, which was vital to creating a micropillar of this scale at a site specific location in a reasonable time. The micropillar was attached to a pin sample holder for further analysis after lift-out.

### Nanoscale X-ray CT

Nanoscale X-ray CT data was acquired on a Zeiss Xradia Ultra 810 instrument. One series of projections was acquired in ‘large field of view’ mode with a 60 s exposure time, 721 projections and a pixel size of 64 nm. Another series of projections was acquired in ‘high resolution’ mode with a 200 s exposure time, 601 projections and a pixel size of 32 nm. The two series were reconstructed with a filtered back projection reconstruction and subsequently combined into a single volume, with the higher resolution data replacing the relevant volume in the lower resolution data. All visualisation and thresholding was performed in the Avizo software package (version 9.0).

The angles between the grain boundaries and the macro-crack were calculated by aligning a slice with the planes of the different boundaries, taking the normal of this aligned slice, and then computing the dot-product with the direction along the axis of the cylinder.

### FIB-SEM processing

A FEI Helios 660 Dual Beam FIB-SEM microscope was used to extract a cross-sectional slab from the pillar sample. The end of the pillar was removed using the FIB at an accelerating voltage of 30 kV and a beam current of 9.4 nA. Subsequently, a 3 μm thick cut was made through the centre of the pillar with the same conditions before attachment to the liftout probe, a cut of the remaining material with a beam current of 2.5 nA and liftout of the pillar section.

The section of the pillar removed using an EasyLift manipulator was attached to a half-moon pronged TEM grid before employing a cleaning cross-section in the FIB at 2.5 nA beam current to polish each face. A block was removed from the cross-sectional slab through a FIB cut along the length of the block at a beam current of 0.4 nA, before attachment of the liftout probe, removal of the remaining material and subsequent liftout. The extracted block was mounted vertically on to a 1 mm diameter pin for a Fischione 2050 tomography holder, before an approximately 2 µm protective Pt cap was deposited. The block was “cleaned” by polishing each face in the FIB, using a beam current of 0.43 nA. Subsequently, rectangular milling of two sides of the block was performed to produce a 1 μm by 1 μm square. Secondary electron images of the sides of the block were recorded at this point in order to match to the nano-CT data in order to perform positional correlation. The finished block underwent an annular milling procedure in which the outer and inner radii were progressively stepped from 2 μm and 1.5 μm to 0.5 μm and 0.15 μm respectively, at a beam current of 80 pA throughout.

### EBSD Analysis

A FEI Helios Xe plasma-FIB was used to prepare a cross-section of the micropillar after the cross-sectional slab was extracted. A cleaning cross-section was employed in the FIB at an accelerating voltage of 30 kV and a current of 59 nA. Subsequently, EBSD mapping was undertaken of the prepared cross-section using an Oxford Instruments NordlysMax detector, employing the SEM at an accelerating voltage of 20 kV and a beam current of 11 nA. Acquisition and data analysis were performed using Oxford Instruments Aztec software (version 3.3).

### STEM Imaging and Tomography

STEM and EDX spectrum imaging were performed using a probe-side aberration-corrected FEI Titan G2 80–200 S/TEM operated at 200 kV. STEM images were collected using a convergence angle of 18 mrad and a high angle annular dark-field (HAADF) detector with an inner angle of 55 mrad. EDX spectroscopy compositional analysis was performed using the Super-X detector system (4 × 30 mm^2^ silicon drift detectors), which possesses a solid angle of ~0.7 srad, using a beam current of ~0.5 nA. For display purposes, all spectrum images were processed using a 3-pixel smoothing window in the Bruker Esprit software.

A STEM-EDX tilt-series was acquired through collection of spectrum images at 5° intervals over a 180° angular range. In the tilt-series a 200 μs per pixel dwell time was employed and the data were summed over 12 frames, to give a total pixel dwell of 2.4 ms. The tilt-series was aligned in the Inspect3D software package, using the extracted Cr Kα maps to determine image shifts and tilt-axis misalignment. The Cr Kα maps were used for alignment as the HAADF series showed little contrast and the Cr Kα maps had a relatively high signal to noise. The simultaneous iterative reconstruction technique (SIRT) algorithm in Inspect3D was used to perform the reconstruction with 20 iterations.

### Data Availability

The datasets generated during and/or analysed during the current study are available from the corresponding author on reasonable request.

## Electronic supplementary material

Supplementary Information
