# Supplementary material for: Multiscale correlative tomography: an investigation of creep cavitation in 316 stainless steel
Source: Sci Rep. 2017 Aug 4;7:7332. doi: 10.1038/s41598-017-06976-5 (PMC5544716; doi:10.1038/s41598-017-06976-5)
Supplement: Supplementary file 1 — Supplementary Information [file 41598_2017_6976_MOESM1_ESM.pdf]

Supplementary Information for:

Multiscale correlative tomography: an investigation of creep cavitation in 316 stainless steel

T. J. A. Slater[a], R. S. Bradley[a], G. Bertali[b], R. Geurts[c], S. M. Northover[d], M. G. Burke[b], S. J. Haigh[b], T. L. Burnett*[a], P. J. Withers[a]

[a] Henry Moseley X-ray Imaging Facility, School of Materials, The University of Manchester, M13 9PL, UK

[b] School of Materials, The University of Manchester, M13 9PL, UK

[c] Thermo Fischer Scientific, FEI Company, Achtseweg Noord 5, Bldg 5651 GG, Eindhoven, The Netherlands

[d] Department of Engineering and Innovation, Materials Engineering, The Open University, Milton Keynes, MK7 6AA, UK

## Correlative Workflow

We have been able to correlate STEM-EDX tomography data with X-ray nanotomography to within a 500 nm x 500 nm x 1 μm volume. The manual registration steps presented describe the process of registration of a STEM tomography data set ( ~ 200 nm in diameter and 1000 nm in length) within an X-ray CT data volume (30 μm in diameter and 100 μm in length).

The sample orientation was tracked throughout the process, with the position of the final STEM tomography nanopillar chosen at a specific location. Post-acquisition registration of the obtained volumes is then used to obtain the final correlated volumes. Spatial correlation of the STEM-EDX tomography and X-ray nanotomography volumes is not possible from the two volumes alone, as features present in the STEM-EDX tomography volume are not visible in the X-ray CT volume due to the limited spatial resolution of X-ray CT. Instead, volume registration is achieved through correlation of the position of SEM images, acquired during the FIB preparation steps, with the X-ray CT volume. Registration of the X-ray CT volume and SEM images is achieved through a manual feature matching, principally related to the cavities decorating the grain boundary (Figure S1).

An SEM image of one face of the extracted cross-sectional slab is initially matched to a slice through the X-ray CT volume (Figure S1). The approximate position of the extracted block can then be determined on the slab through analysis of the position of the sides of the block. SEM images of each face of the block are then manually positioned to match slices in the X-ray CT data via distinctive cavities. In this way, the location of the extracted block can be determined to an accuracy better than 100 nm in all dimensions, positioning the SEM coordinates into that of the X-ray CT coordinate system.

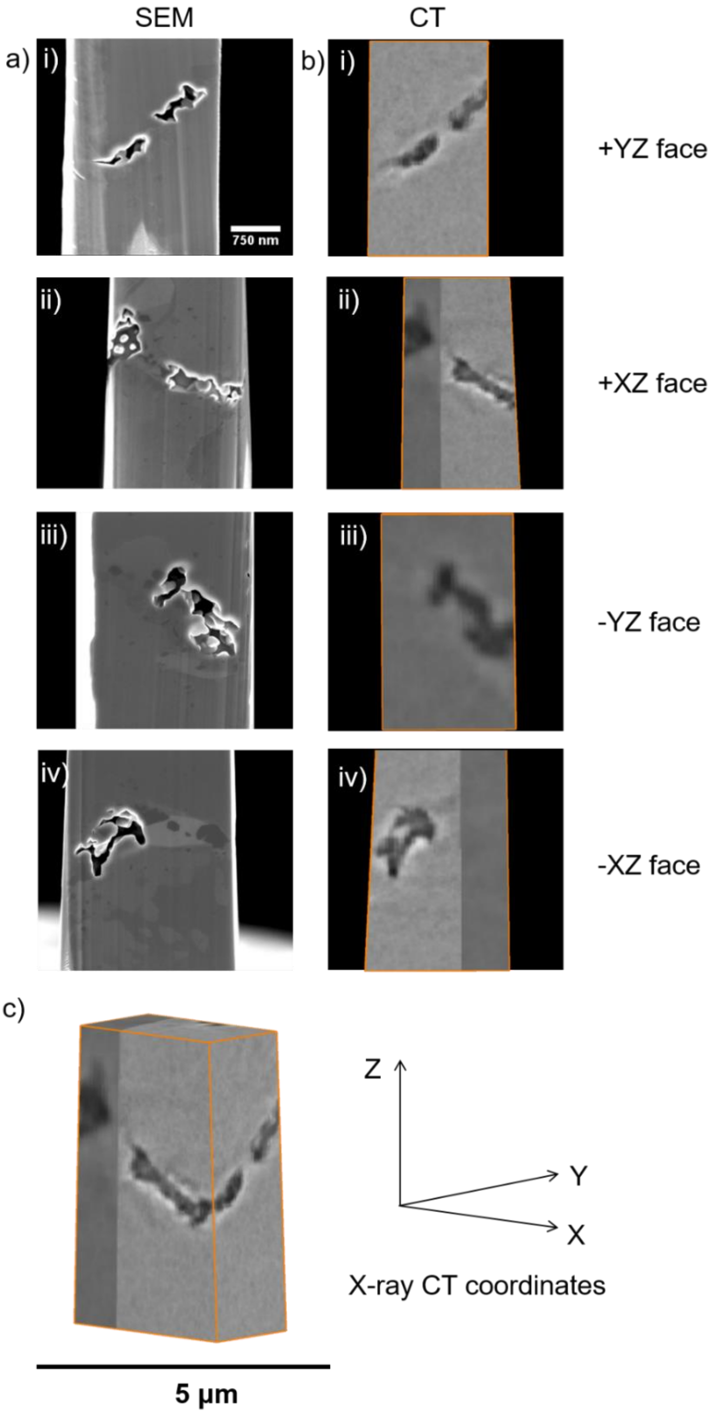


Figure S1. Images displaying the faces of the extracted block and the correlation between a) SEM images and b) slices through the CT data of faces i-iv. c) Faces (i) and (ii) displayed in three dimensions from the CT data with the X-ray CT coordinate system indicated.

Correlation of the position of the STEM nanopillar within the X-ray CT volume relies primarily on the correlation of the block faces. The nanopillar was fabricated in one of the corners of the block, specifically positioned to avoid the cavities in this block, and this positions the nanopillar in an area in the defined X and Y dimensions of less than 500 $nm^2$ in the micropillar. Correlation of the XY position of the nanopillar with that of a top-down view SEM image further increases the positional accuracy to

within a few hundred nanometres (Figure S2.b). The position of the nanopillar in the Z direction of the CT volume is achieved here through positioning the centre of the precipitates at a Z-coordinate in the materials coordinate system that corresponds to the location of precipitates in the correlated SEM image of face (iii). This assumes that the precipitates are located at the same Z-coordinates at the XY position of the needle as they are found at the XY coordinates of face (iii). The rotational transformation of the STEM reconstruction coordinates is determined through correlation of an SEM image of the nanopillar to the STEM-EDX 3D data, through positioning of the higher-Z G-phase.

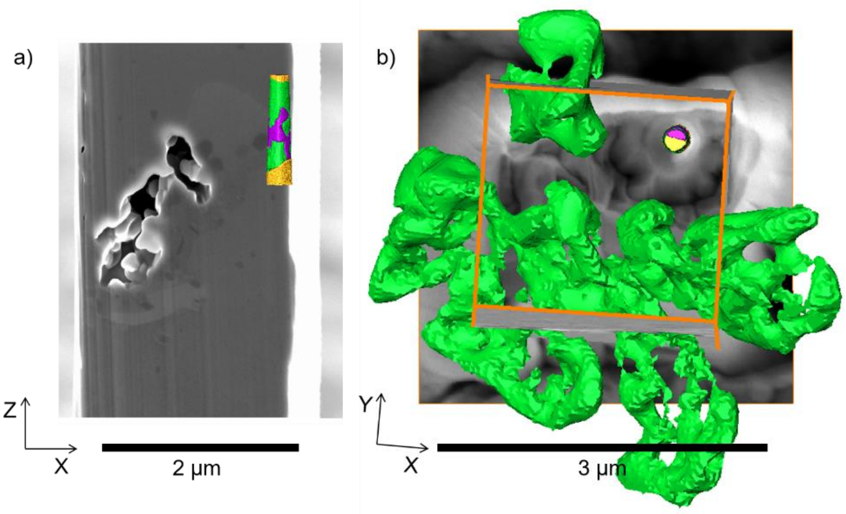


Figure S2. a) Visualisation of the STEM-EDX nanopillar with respect to the SEM image of face (iii) (flipped) and b) with respect to the top-down SEM image taken after annular milling with segmented cavities also shown. The coordinate systems indicated all correspond to the X-ray CT coordinates.
